# Stepwise Covariance-Free Common Principal Components (CF-CPC) With an Application to Neuroscience

**DOI:** 10.3389/fnins.2021.750290

**Published:** 2021-11-11

**Authors:** Usama Riaz, Fuleah A. Razzaq, Shiang Hu, Pedro A. Valdés-Sosa

**Affiliations:** ^1^The Clinical Hospital of Chengdu Brain Sciences, University of Electronic Science and Technology of China, Chengdu, China; ^2^Anhui Provincial Key Laboratory of Multimodal Cognitive Computation, Key Laboratory of Intelligent Computing and Signal Processing of Ministry of Education, School of Computer Science and Technology, Hefei, China; ^3^Cuban Neuroscience Center, Havana, Cuba

**Keywords:** Ultra-high Dimensional Data, Covariance-free, Neuroimaging, EEG, MEG, common principal component (CPC)

## Abstract

Finding the common principal component (CPC) for ultra-high dimensional data is a multivariate technique used to discover the latent structure of covariance matrices of shared variables measured in two or more *k* conditions. Common eigenvectors are assumed for the covariance matrix of all conditions, only the eigenvalues being specific to each condition. Stepwise CPC computes a limited number of these CPCs, as the name indicates, sequentially and is, therefore, less time-consuming. This method becomes unfeasible when the number of variables *p* is ultra-high since storing *k* covariance matrices requires *O*(*kp*^2^) memory. Many dimensionality reduction algorithms have been improved to avoid explicit covariance calculation and storage (covariance-free). Here we propose a covariance-free stepwise CPC, which only requires *O*(*kn*) memory, where *n* is the total number of examples. Thus for *n* < < *p*, the new algorithm shows apparent advantages. It computes components quickly, with low consumption of machine resources. We validate our method CFCPC with the classical Iris data. We then show that CFCPC allows extracting the shared anatomical structure of EEG and MEG source spectra across a frequency range of 0.01–40 Hz.

## Introduction

With exceptional advancements in data acquisition capabilities in recent years, there has been a rise in conducting large-scale neuroscience studies. Increased processing power with the availability of High-Power Computing (HPC) setups gives the neuroscience community ability to compute high-resolution spatial and temporal source imaging and source activity localization, especially in EEG and MEG data. These datasets are gathered with lots of different parameters. These parameters can be different due to age, gender, ethnicity, geographical location, capturing modality, and machine parameters.

Analyzing ultra-high-dimensional neuroimaging data has been time-consuming and challenging. There are many solutions, e.g., Principal Component Analysis (PCA), Independent Component Analysis (ICA), Incremental Principal Component Analysis (IPCA), and sparse incremental Principal Component Analysis (sIPCA) (Yao et al., 2012; Balsubramani et al., 2013; Tang and Allen, 2018; Tabachnick and Fidell, n.d.) developed to overcome high dimensionality. However, only a small number of principal components can explain almost all the variance of a multivariate data and that is where stepwise computation of Principal Components comes. Stepwise PC only a compute a limited number of PCs sequentially. Power method is used to first compute the most dominant PC and then deflation parameter extracts the estimated variance from data (MacKey, 2009; Golub and Van Loan, 1996). Later, the dominant PC is again computed from the remaining data using same procedure, details are in methodology.

However, there are some scenarios when *k* different populations or groups, also known as conditions, are being compared or cross analyzed, and a common latent covariance structure is required. This phenomenon is also known as obtaining common principal components (CPCs). The conventional CPC algorithm was first introduced and studied by Flury (1984). CPC is one of many possible generalizations of standard PCA of several covariance matrices (Jolliffe, 2005). The initial motivation for introducing CPC was to study discrimination problems, where the covariance matrices for different conditions are not equal as required by linear discriminant analysis but more generally share a latent joint principal axis (Flury, 1984; Krzanowski, 1984). The CPC model was mainly criticized because it is essentially a method for simultaneous diagonalization of several positive definite matrices. Rather than a dimensionality reduction method, which is usually the main goal in data analysis (Schott, 1989).

To remedy this (Krzanowski, 1984), proposed a simple, intuitive procedure to estimate an approximation of the CPCs based on the PCA of the pooled sample covariance matrix and the total sample covariance matrix, followed by the comparison of their eigenvectors (Schott, 1989, 1999) proposed an improvement where the latent covariance structure spanned by the first *m* principal components (PC) and their sum is identical for any *k* conditions. This is called Common Subspace Analysis (CSA). These improvements try to achieve the aim of dimensionality reduction. However, CSA still used the inherited concept of finding a common subspace of all groups simultaneously, making it time-consuming and computationally expensive. To remedy this, stepwise CPC was proposed by Trendafilov (2010), which sequentially performs the CPCs functionalities. Stepwise CPC computes fewer latent components, making it computationally less expensive and achieving dimensionality reduction.

The motivation of this study comes from a problem we faced while conducting a previous study where we were trying to decompose the source spectra of EEG and MEG. This decomposition aims to remove pre-identified differences between the two spectra and to develop a transfer function. Estimating common topography between EEG and MEG spectra is one of the elements required for this decomposition process. However, both spectra are ultra-high dimensional, i.e., the number of variables *p* is much larger than the number of observations *n* (Hu, 2020; Riaz, 2021a). To compute a common latent subspace via stepwise CPC, we need the covariance matrix for all *k* conditions, i.e., covariances for EEG and MEG. Since computing covariance requires *O*(*p*^2^) memory which can be time-consuming and even impossible when *p* is large. Thus, conventional stepwise CPC cannot be applied here, as it will require *O*(*kp*^2^) memory space. A covariance-free CPC is required to compute a common latent subspace for ultra-high-dimensional data, which do not compute and save covariance matrix. Some covariance-free methods have been previously proposed to improve other dimensionality reduction methods. For example, IPCA was proposed by Weng et al. (2003) and Yousefi et al. (2017), iterative Kernal PCA proposed by Liao et al. (2010), incremental PCALDA by Dagher (2010), covariance free partial least squares by Jordao et al. (2021). Instead of working with covariance matrices, all these methods achieve dimensionality reduction without computing and saving covariance matrix, which decreases required memory to *O*(*n*).

This article proposes a novel method we call stepwise Covariance-Free CPC (stepwise CFCPC) by merging “covariance free” and common latent subspace concepts. The following sections of the article are a methodology for CFCP then the description of the datasets we are using to test and validate our method. Later we present the results and compare stepwise CPC and stepwise CFCPC for accuracy, computation time, and memory consumption. Furthermore, we lay down the concluding remarks and suggest applications of this improvement.

## Methodology and Materials

### Principal Components Principal Components

PCA is a dimensionality reduction technique that computes Principal Components (PCs) to represent data by linear combination of a significantly less number of vectors. These vectors represent the maximum variance of data that a single vector can represent in a given direction. Other PCs that are orthogonal to their previous PC are computed, and every next PC represents a lesser amount of variance from the previous. Principal components are obtained from the covariance matrix of the data, then eigenvalues and eigenvectors of that covariance matrix are computed for dimensionality reduction (Tabachnick and Fidell, n.d.). For a given data generally, the PCs equal to the number of variables is computed. However, only a small number of PCs represent almost all the data variance, which is why only a few PCs are required. This phenomenon gives birth to the idea of stepwise Principal Components.

### Stepwise Principal Component

Stepwise principal components compute a limited number of PCs and compute them sequentially, which is explicitly required when matrices are of larger sizes. The stepwise PC is obtained by first using the power method to compute the most dominant eigenvalue by normalizing the given matrix, computing the most dominant eigen value, and applying the deflation to extract the variance that has been estimated by λ. It works on a diagonalizable square matrix, which in our case is a covariance matrix *S*_*i*_. The power method iteration algorithm for a given covariance matrix *S*_*i*_ works as shown below. Here μ is the normalized covariance matrix *S*_*i*_, λ is the estimated eigenvector, and *l*_*max*_ are the total number of iteration for convergence. The Power Method Iteration Algorithm defined above gives the largest eigenvalue. The deflation method (MacKey, 2009) omits the estimated covariance to obtain new *S*_*i*_ which is eventually used to compute the next λ. Stepwise-PC repeats this process iteratively for the given number of eigenvalues.


P⁢o⁢w⁢e⁢r⁢M⁢e⁢t⁢h⁢o⁢d⁢I⁢t⁢e⁢r⁢a⁢t⁢i⁢o⁢n

$$\mathrm{for}\,i=1,\ldots ,l_{\max}$$

$$\;\,\mu \leftarrow {\text{S}}_{i}\,\mu$$

$$\;\,\lambda \leftarrow \mu^{T}\,\mu$$

$$\;\,\mu \leftarrow \mu /\sqrt{\lambda }$$

end

D⁢e⁢f⁢l⁢a⁢t⁢i⁢o⁢n

$${\text{S}}_{i}\leftarrow {\text{S}}_{i}-\lambda \,\mu^{T}\,\mu$$

### Conventional Common Principal Component

Conventional CPC is one of the generalizations of PCA for *k* conditions and their covariance matrices, as mentioned in Flury (1984). In CPC, it is assumed that all *k* conditions have the same mean, and their covariance matrices *S*_*i*_ are all positive definite and diagonalizable.


(1) $$H_{C\,P\,C}\,:\,{\text{Q}}^{\text{T}}\,{\text{S}}_{i}\,\text{Q}={\text{D}}_{i}^{2},i=1,2,\ldots ,k$$

Here **Q** is the common orthogonal matrix for all conditions and ${\text{D}}_{i}^{2}$ is the positive orthogonal matrix for each condition. CPC estimations find the common eigenvectors and eigenvalues for covariance matrices **S**_*i*_ for all *k* conditions and *n*_*i*_(> *p*) degrees of freedom or number of observations such that:


(2) $${\text{S}}_{i}\approx {\text{QD}}_{i}^{2}\,{\text{Q}}^{\text{T}}$$

CPC computes the latent components all at once by computing maximum likelihood components of parameters **Q** and ${\text{D}}_{i}^{2},i=1,2,\ldots ,k$ using the following optimization problem of minimizing negative log-likelihood as mentioned in Flury (1984).


$$\mathrm{Minimize}\,\sum_{i=1}^{k}n_{i}\,\left[\log\left(\det\left({\text{QD}}_{i}^{2}\,{\text{Q}}^{\text{T}}\right)\right)+t\,r\,a\,c\,e\,{\left({\text{QD}}_{i}^{2}\,{\text{Q}}^{\text{T}}\right)}^{-1}\,{\text{S}}_{i}\right]$$

(3) $$\;=\sum_{i=1}^{k}n_{i}\,\left[\log\left(\det\left(t\,e\,x\,t\,b\,f\,D_{i}^{2}\right)\right)+t\,r\,a\,c\,e\,\left({\text{D}}_{i}^{-2}⊙\left({\text{Q}}^{\text{T}}\,{\text{S}}_{i}\,\text{Q}\right)\right)\right]$$

(4) $$\mathrm{Subject}\mathrm{to}\;\left(\text{Q},{\text{D}}_{1},{\text{D}}_{2},.\ldots ,{\text{D}}_{k}\right)\in \vartheta \left(p\right)\times D{\left(p\right)}^{k},$$

Where ⊙ is the Hadmard product, the Lie group of all *p*×*p* orthogonal matrices is denoted by ϑ(*p*) and $D\,{\left(p\right)}^{k}=\underset{k}{\underbrace{D\,\left(p\right)\times \ldots \times D\,\left(p\right)}}$. Here D(*p*) is the linear subspace of all *p*×*p* diagonal matrices. However, to satisfy the first-order optimality condition for a stationary point of CPC objective function, $\sum_{i=1}^{k}n_{i}\,{\text{Q}}^{T}\,{\text{S}}_{i}\,{\text{QD}}_{i}^{-2}\,\,i\,s\,\,s\,y\,m\,m\,e\,t\,r\,i\,c$ and $d\,i\,a\,g\,\left({\text{Q}}^{T}\,{\text{S}}_{i}\,\text{Q}\right)={\text{D}}_{i}^{2}$ for all *k* + 1 conditions simultaneously. After submitting these values in Equation (3), we can define CPC estimation as the following likelihood problem.


(5) $$\mathrm{Minimize}\;\sum_{i=1}^{k}n_{i}\,\log\left(\det\left(d\,i\,a\,g\,\left({\text{Q}}^{\text{T}}\,{\text{S}}_{i}\,\text{Q}\right)\right)\right)$$

(6) Subject⁢to Q∈ϑ⁢(p)

Here **Q** is the set of all orthogonal matrices that contain all the CPCs, which are computed simultaneously. This is a basically FG diagonalization solution replacing the original problem mentioned in Equations (3) and (4). CPC works efficiently for covariance matrices **S**_*i*_ that are positive definite and positive semi-definite as well.

### Stepwise Common Principal Component

Stepwise CPC is performed by imitating the standard PCA to achieve dimensionality reduction, i.e., finding the latent components one after another (Trendafilov, 2010). It does not compute all the CPCs at once, and instead, it computes only a desired number of CPCs sequentially. First, it transforms the CPC problem into a vectorize form and then solves the *p* identical problems expressed in Equations (7) and (8) sequentially.


(7) $$\mathrm{Minimize}\;\sum_{i=1}^{k}n_{i}\,\log\left(q^{\text{T}}\,S_{i}\,q\right)$$

(8) $$\mathrm{Subject}\,\mathrm{to}\;q^{\text{T}}\,q=1$$

Here *q* are the common PCs computed sequentially. Stepwise CPC is done by finding the first CPC which will be *q*_*p*_ which will give a minimum of Equation (7) in the unit sphere in *ℝ^p^*, then the next CPC is found that will be *q*_*p*−1_ which will give a minimum of Equation (7) in the unit sphere in *ℝ^p^* and it will be orthogonal to *q*_*p*_. So, each minimum found using this idea will be greater than the previous one as it is found in the orthogonal domain of the previous minimization domain. Since the quantities *q*^T^*S*_*i*_*q* are bounded by the smallest and largest eigenvalues of **S**_*i*_, the objective function in Equation (7) will be bounded on a unit sphere *ℝ^p^*. However, for the purpose of dimensionality reduction, it is more feasible to obtain the CPCs in reverse order, i.e., representing all CPCs by a variational eigenvalues definition represented in Hord and Johnson (2012) as **Q**_*p*_ = [*q*_1_,*q*_2_,…,*q*_*p*_] which *p*×*p* orthogonal matrix containing the CPCs obtained from Equation (7) and (8). So the *jth* CPC can be shown by the optimization problem shown in Equations (9) and (10).


(9) $$\mathrm{Minimize}\;\sum_{i=1}^{k}n_{i}\,\log\left(q^{\text{T}}\,S_{i}\,q\right)$$

(10) $$\mathrm{Subject}\,\mathrm{to}\;q^{\text{T}}\,q=1\,\mathrm{and}\,q^{\text{T}}\,{\text{Q}}_{j-1}=0_{j-1}^{\text{T}}$$

Where **Q**_*j*−1_ = [*q*_1_,*q*_1_,…,*q*_*j*−1_] and the *jth* CPC can be obtained by solving *j* problems similar to the ones shown in Equations (9) and (10). This eventual solution will be similar to the one computed in the original CPC shown in Equations (5) and (6) but has the feature that it can be stopped at any point that is 1 ≤ *j* ≤ *p*. To compute the next eigenvector, one can use an already computed **Q**_*j*_ and do not need to compute all the eigenvectors from the start. The first-order optimality conditions for Equations (9) and (10) can be resolved by Theorem 3.1 in Trendafilov (2010) study. The equation resolves to obtain the first-order optimality condition is shown in Equation (11).


(11) $$\Pi_{j}\,\left(\sum_{i=1}^{k}\frac{n_{i}\,{\text{S}}_{i}}{q_{j}^{\text{T}}\,{\text{S}}_{i}\,q_{j}}\right)\,q_{j}=0_{p\times 1}$$

Where **Π***_j_* the deflation parameter or projector ${\text{I}}_{p}-B_{j}\,{\text{Q}}_{j}^{\text{T}}$ and $\sum_{i=1}^{k}\frac{n_{i}\,S_{i}}{q_{j}^{\text{T}}\,S_{i}\,q_{j}}$ is the gradient of CPC objective function from Equation (9). The theorem solves the first-order optimality condition and gives a general case as shown in Equation (13) and for *j* = 1 as shown in Equation (12).


(12) $$\left[\left(n_{1}\,\frac{{\text{S}}_{1}}{q_{1}^{\text{T}}\,{\text{S}}_{1}\,q_{1}}+\cdots +n_{k}\,\frac{{\text{S}}_{k}}{q_{1}^{\text{T}}\,{\text{S}}_{k}\,q_{1}}\right)-n\,{\text{I}}_{p}\right]\,q_{1}=0_{p\times 1},$$

For *j* = 2,…,*p*


(13) $$\left[\left({\text{I}}_{p}-{\text{Q}}_{j-1}\,{\text{Q}}_{j-1}^{\text{T}}\right)\,\left(n_{1}\,\frac{{\text{S}}_{1}}{q_{j}^{\text{T}}\,{\text{S}}_{1}\,q_{j}}+\cdots +n_{k}\,\frac{{\text{S}}_{k}}{q_{j}^{\text{T}}\,{\text{S}}_{k}\,q_{j}}\right)-n\,{\text{I}}_{p}\right]\,q_{j}=0_{p\times 1}.$$

Equations (12) and (13) show that the CPCs can be computed by solving *p* symmetric eigenvalues problems. Trendafilov (2010) solves this problem by using a modified version of the standard power method to solve the eigenvectors and eigenvalues problem to compute the CPCs defined by Equations (12) and (13). The *Power Method Iteration* algorithm in section “Stepwise Principal Components” is used to compute the CPCs iteratively. The modification is that this algorithm updates the covariance matrix at each step. In particular, this is an algorithm of gradient ascend category—Algorithm 1 in Appendix where the indices of the power iteration are given in the parenthesis. The resultant vector *q*_*j*_,*j* = 1,2,…*p* from Algorithm 1 is the CPCs. A small number of iterations (even less than 3) are required if the eigenvectors are well separated.

#### Stepwise Common Principal Component Implementation

Stepwise, CPC implements (Equations 4–7) by taking the covariance matrices *S*_*i*_ for *k* conditions, the number of common latent PCs to be computed is *p*_*max*_, and *l*_*max*_ is the number of iterations required for convergence. Refer to the Algorithm 1 in Appendix. The stepwise CPC algorithm computes only a specific number of CPCs. As output, it gives eigenvectors **Q***_pmax_* for common latent sub-space under all *k* conditions along with λ*_pmax_* the eigenvalues for each particular condition. However, the problem with stepwise CPC arises when the number of variables *p* becomes too large.

### Stepwise CF-CPC

The idea behind covariance free stepwise CPC is to not compute the covariance at any step of the algorithm. To achieve this purpose, we propose that instead of calculating the covariance, replace it with its mathematical definition of covariance and apply this concept in the basic Algorithm 1 of stepwise CPC. This can be done by expanding the covariance formula, as shown in Equation (14).


(14) $${\text{S}}_{i}=\frac{\left({\text{H}}_{i}\,{\text{X}}_{i}^{\text{T}}\right)\,\left({\text{H}}_{i}\,{\text{X}}_{i}\right)}{n_{i}}$$

Here **H** is the average reference when applied to the will replicate the subtraction of mean from the data that is used conventionally to compute covariance, which is by definition ${\text{H}}_{i}={\text{I}}_{i}-\,\frac{1_{i}^{\text{T}}\,1_{i}}{n_{i}}$ and **X**_*i*_ is actual data for which the covariance was computed for stepwise CPC. Equation (14) can also be written as Equation (15).


(15) $${\text{S}}_{i}={\text{W}}_{i}^{\text{T}}\,{\text{W}}_{i}$$

Here **W**_*i*_ can be defined as $\frac{H_{i}\,X_{i}}{\sqrt{n_{i}}}$ making Equation (14), as shown in Equation (15).

Using this technique, we can achieve the same results while simplifying and changing the computations and formulations to make it covariance-free, requiring *O*(*kn*) memory for *k* conditions where *n* < < *p*. Stepwise-CFCPC is fast, memory efficient, and will be optimal for ultra-high dimensional data.

#### Stepwise CF-CPC Implementation

Algorithm 2 (refer to Appendix) explains the flow of how stepwise CF-CPC works. We are trying to achieve this algorithm to make it covariance-free and optimize it in terms of computations and memory usage. The inputs *p*_*max*_,*l*_*max*_*andn* are the same as for the original stepwise-CPC, i.e., the following are the steps we have taken for optimization purposes:

• Algorithm 2 takes data **X**_*i*_ as input instead of the covariances **S**_*i*_, which saves memory and computation power.
• We perform singular value decomposition (SVD) (Klema and Laub, 1980) instead of estimating eigenvectors (Andrew, 1973).

(16) $$\Pi_{i}={\text{I}}_{p}-\sum_{a=1}^{j-1}q_{a}\,q_{a}^{\text{T}}$$

for _*j* = 1,…,*p*_*max*__

• Furthermore, we replace the deflation parameter **Π***_j_* with its mathematical definition as Equation (9) (MacKey, 2009).
• We substitute the sum of covariances with a matrix formed when the definition of covariance is used from Equation (14).

Initially, the stepwise CPC was implemented for an R-package named “cpca” (Ziyatdinov et al., 2014). Since our working environment is MATLAB 2018b (The MathWorks, n.d.), we implemented and verified the stepwise CPC in MATLAB and the same for stepwise CFCPC.

### Materials

To test the covariance-free version of stepwise CPC, we used two datasets. The first data we analyzed (for validation purposes) was Fisher’s IRIS data (Anderson, 1935; Fisher, 1936), and this is the same data that was analyzed in the original stepwise CPC algorithm (Trendafilov, 2010). Secondly, we have analyzed neuroscience data.

#### Iris Data

We tested our stepwise CF-CPC algorithm initially on Fisher’s IRIS data. The purpose is to validate if the covariance-free stepwise CPC gives the same results as stepwise CPC. Fisher IRIS data has 150 examples/observations and four variables, making it a [150×4] matrix. To transform the data into *k* = 3 conditions, we divided that data into chunks of 50 examples with four variables in each chunk, making three matrices of size [50×4]. Later we tested both stepwise CPC and stepwise CFCPC on this data for validation purposes.

#### MEEG Data

The neuroimaging data we are using comprises source spectra of two modalities, electroencephalography (EEG) and magnetoencephalography (MEG), with 45 subjects in each group. EEG subjects were picked from an extensive database of the Cuban Human Brain Mapping project (CHBM) (Valdes-Sosa et al., 2021). MEG data of a similar sample size of 45 were picked from Human Connectome Project (HCP) (WU - Minn Consortium Human Connectome Project, 2017). The EEG data we used was source spectra computed from a novel inverse solution BC-VARETA (Gonzalez-Moreira et al., 2018; Paz-Linares et al., 2018). The size of the source spectra [8002×80×45] is [*sources*×*frequency*×*examples*]. Here the first dimension representing the number of brain sources/generators. The second dimension is the number of frequency points (in this case, there are 80 frequency points with a step-size of 0.5 Hz, so the total analyzed source spectra was 0–40 Hz), and the third dimension represents the number of subjects involved in the study. MEG source spectra were computed using the same inverse solution used of EEG data, and the size of the source spectra matrix was also the same. Before computing the common principal spectral component, we scaled data and log-transformed it for visualization. Since we are analyzing 8002 sources at each of the 80-frequency points, we rearranged the source spectra to convert them into a 2D array of size [45×640160]. There is a total of 640160 variables, with each group of 8002 variables representing one frequency point. So, the inputs go into both algorithms of stepwise CPC are *p* = 8002×80 = 640160, [45 45] and k =  2 where *p* > > *n*.

### Stepwise Common Principal Component and Stepwise CFCPC Computation

We computed the CPC subspace score along with eigenvalues for each modality using both algorithms. The same dataset and machine specifications are used to compare the results in terms of time consumed to compute the CPC. The specifications are explained below:

• Intel(R) Core (TM) i5-7200U CPU @ 2.50GHz 2.70 GHz
• 16.0 GB DDR3 RAM
  Windows 10–64-bit operating system, x64-based processor
• MATLAB 2018b (The MathWorks, Inc, 1994-2021)

We observed that computing covariance for CPC above a certain number of variables is impossible on this machine because of “out of memory” issues. Additionally, there is a limit for the number of variables on which computing covariance was successful. However, inside the stepwise CPC algorithm, it returns the error of running out of memory. So, we tested both algorithms for a specific number of variables to check two things:

• What is the limit of the number of variables for both algorithms?
• How much time each algorithm takes to compute a certain number of variables.

Once we have verified the number of variables for which the CPCs are successfully computed, we computed the CPCs for the MEEG data discussed above. These CPCs will be the shared space eigenvectors for the sources spectra captured using two different modalities, i.e., EEG and MEG. Once these CPCs are computed, we applied the eigenvalues on the shared common subspace to visualize the common space generated for EEG and MEG, respectively. In the end, we compared the original EEG and MEG spectra with the respective estimated common subspace for accuracy.

## Results

**FIGURE 1:**
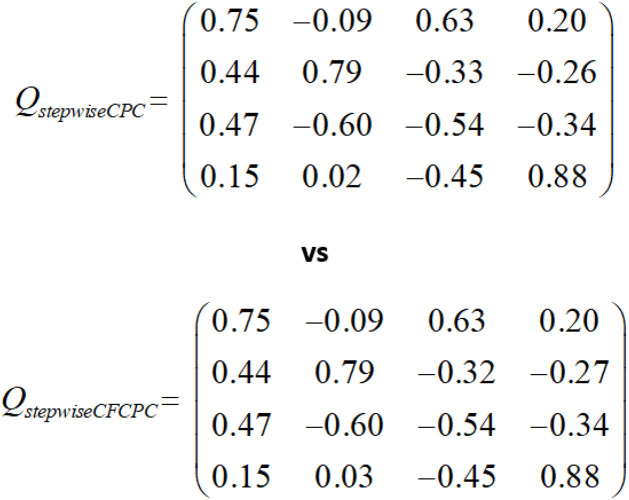
Stepwise CPC vs. covariance free stepwise CPC.

As mentioned earlier, we need to compute covariance first and input that to the algorithm for stepwise CPC. For stepwise CF-CPC, pass the iris data as it is. We computed four CPCs *Q* and their scale factor λ for both versions of stepwise CPC. The results show that values for all four outcomes of both *Q* and λ for Algorithm 1 and Algorithm 2 are the same as shown in Figure 1.

Next, we used neuroscience data as explained in materials sections and applied both algorithms to look for time and memory consumption results. To compute one CPC with both methods, we keep the values for *p*_*max*_ = 1,*l*_*max*_ = 1& *n* = [45 45] for both algorithms. We recorded computation time and looked for “out of memory” errors for a series of variables selected from the EEG and MEG datasets and recorded the results in the form of Table 1. It is evident in the table that stepwise CFCPC works smoothly on all different sets of variables from 100 till 640160, not giving any memory errors, and the execution time is very nominal. However, stepwise CPC observes an exponential rise in the execution time when the number of variables is increased. Additionally, stepwise CPC could not compute a single CPC beyond 15,000 variables, and covariance computation was not successful beyond 25,000 variables on the defined system specifications.

**TABLE 1:** Comparison of stepwise CPC and stepwise CFCPC (execution time and memory consumption comparison). Successful computation of covariance for a different number of variables. Success or failure in execution of Algorithm 1 and 2 with execution time for the different number of variables.

**Method**	**Variables**	**Covariance computation**	**Algorithm execution**	**Time consumed**
Stepwise CPC	100	Success	Success	0.104310s
	500	Success	Success	0.588175s
	1,000	Success	Success	3.158705s
	5,000	Success	Success	441.268313s
	8,002	Success	Success	2000.196520s
	15,000	Success	Success	6607.873001s
	25,000	Success	Failed	NA
	50,000	Failed	Failed	NA
	100,000	Failed	Failed	NA
	200,000	Failed	Failed	NA
	400,000	Failed	Failed	NA
	640,160	Failed	Failed	NA
Stepwise CFCPC	100	Success	Success	0.011920s
	500	Success	Success	0.035195s
	1,000	Success	Success	0.056952s
	5,000	Success	Success	0.507487s
	8,002	Success	Success	0.746992s
	15,000	Success	Success	1.378456s
	25,000	Success	Success	2.206656s
	50,000	Success	Success	4.378850s
	100,000	Success	Success	8.402318s
	200,000	Success	Success	16.705613s
	400,000	Success	Success	34.466983s
	640,160	Success	Success	65.797402

**FIGURE 2:**
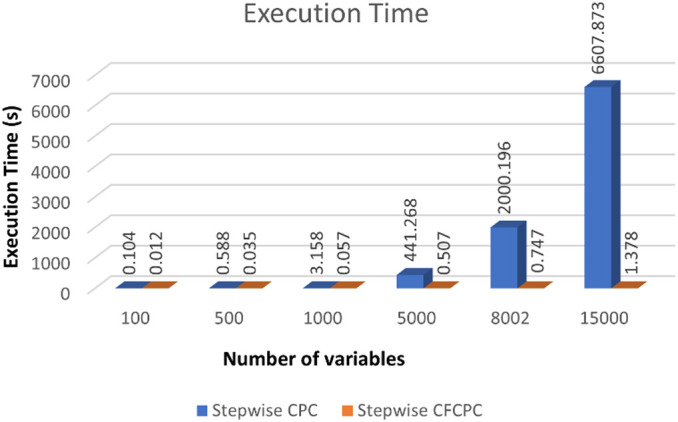
Execution time comparison between stepwise CPC and stepwise CFCPC. Blue bars represent stepwise CPC, and orange representing stepwise CFCPC. Execution time is shown on the y-axis and x-axis, representing the number of variables.

**FIGURE 3:**
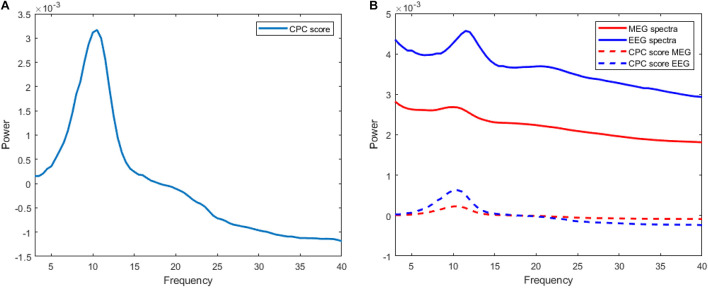
**(A)** Curve representing common source topography Q computed via stepwise-CFCPC with scale value (λ) 0.0072735 for MEG and 0.19906 for EEG. Frequency is on the x-axis, and the y-axis represents the power scale. **(B)** Comparison of actual MEG and EEG source spectra with their CPC-scored versions.

The trend of execution time for both algorithms can be visualized as shown in Figure 2. Stepwise CFCPC consumes less time than stepwise CPC even when the number of variables is low. This execution time remains stable when the number of variables is increased from 100s to 1,000s for stepwise CFCPC. However, stepwise CPC resulted in an exponential increase in computation time as variables are increased. It took almost 5,000-folds more time for the maximum number of variables it could compute CPC successfully. We visualized the computation of CPC for EEG and MEG data for its accuracy and interpreting the source spectra of both modalities. The purpose of computing CPC for these source spectra is to compute a common source topography captured by both modalities in different conditions and find a scale factor or eigenvalue assigned to each modality’s source spectra to compute its respective common topography. These common topography and scale factors will be used as an ingredient in another study to identify and remove the differences between the source spectra of EEG and MEG (Riaz, 2020). A visualization of how this common topography is acquired is shown in Figure 3A. The common source topography represents common topographical features of spectra when visualized in the frequency domain. One of the clear components is the alpha peak visible in the alpha band (7-12 Hz). The computed CPC for common topography has the common subspace eigenvectors and eigenvalues for EEG and MEG spectra. These eigenvalues applied on the computed common subspace estimate the common topography scores for EEG and MEG, as shown in Figure 3B. The solid lines are the original EEG and MEG spectra, whereas the dotted lines show the latent estimated common topography for each modality. This estimated common topography is obtained by applying the scale factor or eigenvalue λ to the common topography *Q* shown in Figure 3A.

The estimated common topographies shown in dotted lines depict that CPC has successfully computed the desired common latent subspace of common topography for each modality. The difference in the original spectra and estimated common topography can be explained because we are only estimating one of the few elements from which the complete source spectra is composed. This common latent subspace computed for common topography between EEG and MEG source spectra is the baseline for the transferal of source spectra between EEG and MEG, as discussed in Riaz (2020) and Riaz (2021b). In the mentioned studies, we are trying to create a transfer function from EEG to MEG source spectra and vice versa. We decompose each spectra into three components, i.e., Common Topography with scale values (computed using the algorithm of this study), Xi-process, and individual differences. These elements are used to synthetic MEG source spectra from EEG and vice versa.

## Discussion

In this study, we developed stepwise CFCPC for computing common stepwise subspace in ultra-high dimensional data. First, we validated results stepwise CPC and CFCPC and found that the computation of *Q* and λ with this new method is similar to stepwise CPC. Once our proposed improvement is validated, we tested the neuroimaging dataset (source spectra from two popular databases, i.e., EEG from CHBM and MEG from HCP) for a single CPC to compare the efficiency of both algorithms. The common topographical subspace obtained for EEG and MEG source spectra can be used as a baseline to analyze the phenomenon that the captured source spectra with EEG and MEG pose the same information gathered in different scenarios, conditions, or settings. However, when we try to compute a single CPC with conventional stepwise CPC, it turns out to be highly computationally expensive. The computationally expensive nature of stepwise CPC is evident in Table 1 as well that as the number of variables is increased from 100s to 1,000s, the execution time is increased a lot. However, this is not the case when we used our proposed CFCPC to compute a single CPC. An increase in the number of variables did not affect the computation time too much. In fact, for the maximum number of variables, 640160 CFCPC took just beyond a minute to compute a single CPC, which is not the case for stepwise CPC, which failed to compute CPC beyond 15,000 variables. These results prove our initial assumption that computing covariance for ultra-high dimensional data can be computationally expensive and takes a substantial amount of memory. It is also evident from Table 1 that beyond 15,000 variables, computing covariance took the system to out of memory error. In stewpsie CFCPC, we did not encounter any out-of-memory error, which we are trying to achieve using this algorithm.

There is a rise in the processing and dimensionality reduction in high dimensional datasets with exponential data gathering capabilities. Stepwise CFCPC can be a global solution when computing one or multiple CPCs, and computing covariance can be an issue. The issue of computing covariance for ultra-high dimensional data can occur in other areas of neuroscience (raw EEG, MEG data, neurogenetics Data (Genome-Wide Association Study of Parkinson’s Disease: Genes and Environment, n.d.) and many other fields like microarrays in genetics, data from an array of sensors to capture geological or astronomical data, etc. (Bonham-Carter et al., 1990; Pesenson et al., 2010; Alonso-Betanzos et al., 2019). In repetitive resampling like bootstrap, where multiple CPCs are required, we can use stepwise CF-CPC. In this study, we have analyzed 640160 variables which is ultra-high dimensional data. Even most variables in genetics are less than the number of variables handled in this study which tells us that the application of this covariance-free stepwise CPC is valid even in genetics and another ultra-high dimensional datasets.

This version of stepwise CFCPC has its applications in neuroimaging, where CPCs are used to extract common features or principal components of multivariate data. Li (2016) in his study used CPC to perform classification on a multivariate time series EEG data for different clusters obtained from the original time series. Similarly, extracting common spatial patterns (CSP) from multivariate EEG data using CPC is achieved in many studies involving Brain-Computer Interface (BCI) (Wei et al., 2005; Wang and Wu, 2008; Meisheri et al., 2018). Similarly, another study conducted interpretable principal components analysis on multilevel multivariate functional data to decompose total variation into subject-level and replicate-within-subject level (i.e., electrode-level) variation. This decomposition provides interpretable components that can be sparse among variates (e.g., frequency bands) and have localized support over time within each frequency band (Zhang et al., 2019). All these studies use CPC to compute their PCs; CSPs from multivariate data with high dimensions and stepwise CFCPC can help resolve these multivariate problems in a lesser amount of time which is especially essential in the case of BCI.

Similarly, in the domain of genetics, where the data is also multivariate and high dimensional, principal components are often required for dimensionality reduction in data analysis. Many studies estimate the time-scale for the evolution of additive genetic variance-covariance matrices (G-matrices), which is a crucial issue in evolutionary biology and genetics (Arnold and Phillips, 1999; Steppan et al., 2002; Conner et al., 2003; Mezey and Houle, 2003; Cheverud and Marroig, 2007). This comparison is also essential to see if different populations have the same genetic structure. This comparison of variances-covariance, i.e., G-matrices, is a high-dimensional problem and is often done using CPCs. Stepwise, CFCPC can help optimize these problems in terms of time and resources.

## Conclusion

We propose a covariance free improved version of Stepwise CPC initially proposed by Trendafilov (2010). Computing covariance in ultra-high dimensional data causes the failure of the original algorithm as it consumes too much time and goes out of memory error for a large number of variables. The motivation for this study was an analysis of MEEG data where the traditional approaches fail. In contrast, our proposed stepwise CFCPC can work efficiently for ultra-high dimensional data while consuming very minimal memory and taking a small amount of time. Stepwise-CFCPC can be a global solution for computing CPCs for datasets in neuroscience (Areshenkoff et al., 2021), bioinformatics (Foo et al., 2017), gene microarray (Alonso-Betanzos et al., 2019), and multisensory node systems (Bonham-Carter et al., 1990; Pesenson et al., 2010). The improvements in the current algorithm can be finding the optimal number of CPCs to be computed. Currently, we are giving required CPCs to be computed as per data requirements. Further improvements can be made by applying the improvements for CPC analysis suggested in Fernández-Albert et al. (2014), Duras (2020), and Bagnato and Punzo (2021). Another direction for future analysis can be implementing stepwise CFCPC with sparsity conditions.

## Data Availability Statement

Publicly available datasets were analyzed in this study. This data can be found here: CHBM, https://www.nature.com/articles/s41597-021-00829-7; HCP, https://www.humanconnectome.org/storage/app/media/documentation/s1200/HCP_S1200_Release_Reference_Manual.pdf.

## Ethics Statement

The studies involving human participants were reviewed and approved by CNEURO, Cuba for CHBM and NIH, United States for HCP data. The patients/participants provided their written informed consent to participate in this study.

## Author Contributions

PV-S gave the conception and design of the study, supervised the development, experimentation, and writing of the manuscript. UR and FR developed and implemented stepwise-CFCPC algorithm, performed the experiments, wrote the manuscript from first to final draft. SH changed the CPCA R-package to MATLAB script. All authors contributed to manuscript revision, read, and approved the submitted version.

## Conflict of Interest

The authors declare that the research was conducted in the absence of any commercial or financial relationships that could be construed as a potential conflict of interest.

## Publisher’s Note

All claims expressed in this article are solely those of the authors and do not necessarily represent those of their affiliated organizations, or those of the publisher, the editors and the reviewers. Any product that may be evaluated in this article, or claim that may be made by its manufacturer, is not guaranteed or endorsed by the publisher.

## Figures and Tables

**Appendix A1:** Table explaining symbols used in paper and their description.

**Symbol**	**Description**
*Q*	Set of computed for CPCs
*k*	Number of conditions or groups
*S*_*i*_	Covariance matrix
$D_{i}^{2}$	Diagnosable Orthogonal matrices
*p*	Total number of variables
*n*	Total number of observations
*I*_*p*_	Identity matrix for deflation
λ	Number of eigenvalues
*p*_*max*_	Limited number of CPCs computed
*l*_*max*_	Maximum number of iterations for convergence
**Π**	Deflation parameter
*q*	Single common CPC
*X*_*i*_	Dataset captured in k different conditions

## Appendix

**Algorithm 1**

**Algorithm 2**
